# Important Decision of the Supreme Court on Imitations

**Published:** 1883-12

**Authors:** 


### IMPORTANT DECISION OF THE SUPREME COURT ON IMITATIONS.

In July, 1882, the Supreme Court of Rhode Island, by Final Decree, enjoined William H. Hughes, Theodore S. Hughes, and the Hughesdale Manufacturing Company from offering for sale "Acid Phosphate," so-called, in any package which shall be a substantial or colorable imitation of Horsford's Acid Phosphate.

Sept. 24, 1883, the Court decided that William H. Hughes and Theodore S. Hughes had violated said Injunction by selling the "Hughes Acid Phosphate," so-called, and that a writ of attachment must issue against them, whereupon the said respondents were fined $300 each.

The Rumford Chemical Works again warn all persons from selling any imitation of Horsford's Acid Phosphate, or any Acid Phosphate under any style of label, or form of package, for use as a medicine or article of diet, or ingredient to be employed in beverages or food; or any proprietary article for use as a medicine or beverage, under the name of "Acid Phosphate," as they will thereby infringe their trade-mark, copyright, or patents, and render themselves liable for damages.

With reference to the above, it affords us pleasure to note the prompt and vigorous action taken by the Rumford Chemical Works in this case, and the recognition by the Supreme Court of the rights of said corporation. The disreputable and growing practice of infringing upon well-known and meritorious articles, after they have acquired reputation, popularity and value, should be discountenanced by all respectable dealers, as it is by the Courts, and the attempts of unscrupulous persons to build up a business upon the capital of somebody else, and thus perpetrate a willful fraud upon the community, should be everywhere condemned; and the trade, moreover, should do all in their power to protect the rights of individuals and corporations in the exercise of a legitimate business.

